# Generation and quantitative proteomics analysis of CK2α/α’^(−/−)^ cells

**DOI:** 10.1038/srep42409

**Published:** 2017-02-17

**Authors:** Christian Borgo, Cinzia Franchin, Stefano Scalco, Valentina Bosello-Travain, Arianna Donella-Deana, Giorgio Arrigoni, Mauro Salvi, Lorenzo A. Pinna

**Affiliations:** 1 Department of Biomedical Sciences, University of Padova, Via U. Bassi 58/B, Padova, Italy; 2 Proteomics Center, University of Padova and Azienda Ospedaliera di Padova, via G. Orus 2/B, Padova, Italy; 3 Department of Molecular Medicine, University of Padova, Via Gabelli 63, Padova, Italy; 4 CNR Institute of Neurosciences, Via U. Bassi 58/B, Padova, Italy

## Abstract

CK2 is a ubiquitous, constitutively active, highly pleiotropic, acidophilic Ser/Thr protein kinase whose holoenzyme is composed of two catalytic (α and/or α’) subunits and a dimer of a non-catalytic β subunit. Abnormally high CK2 level/activity is often associated with malignancy and a variety of cancer cells have been shown to rely on it to escape apoptosis. To gain information about the actual “druggability” of CK2 and to dissect CK2 dependent cellular processes that are instrumental to the establishment and progression of neoplasia we have exploited the CRISPR/Cas9 genome editing technology to generate viable clones of C2C12 myoblasts devoid of either both the CK2 catalytic subunits or its regulatory β-subunit. Suppression of both CK2 catalytic subunits promotes the disappearance of the β-subunit as well, through its accelerated proteasomal degradation. A quantitative proteomics analysis of CK2α/α’^(−/−)^ versus wild type cells shows that knocking out both CK2 catalytic subunits causes a rearrangement of the proteomics profile, with substantially altered level ( > 50%) of 240 proteins, 126 of which are up-regulated, while the other are down-regulated. A functional analysis reveals that up- and down-regulated proteins tend to be segregated into distinct sub-cellular compartments and play different biological roles, consistent with a global rewiring underwent by the cell to cope with the lack of CK2.

CK2 (an acronym derived from the former misnomer “casein kinase 2”) denotes a ubiquitous, highly conserved, acidophilic Ser/Thr protein kinase whose holoenzyme is composed by two catalytic subunits (α and/or α’) tightly associated with the dimer of a non-catalytic β subunit^1^,^2^,^3^,^4^,^5^. CK2 is one of the most pleiotropic protein kinases, with hundreds of known substrates and seemingly responsible for the generation of a large proportion of the human phosphoproteome^6^,^7^, being implicated in a myriad of cellular functions. Its participation in signalling pathways however is not straightforward and “hierarchical” as in the case of many other kinases implicated in phosphorylation cascades, as CK2 is active in the absence of either specific agonists/stimuli or phosphorylation by other kinases^8^. Nevertheless CK2 critically affects the whole network of cellular signalling operating in a “lateral” rather than “vertical” manner^9^.

CK2 has been associated with a wide spectrum of global diseases^3^,^10^,^11^,^12^,^13^ with special reference to cancer^9^,^14^. Its mode of implication in neoplasia however is different from that of the majority of “onco-kinases”. These latter generally are the products of oncogenes whose mutation gives rise to kinases that have lost the property of becoming active only in response to specific stimuli and which thereafter display unscheduled activity also under basal conditions. Quite often oncokinases are responsible for the perturbation of individual signalling pathways and their targeting is proving a valuable tool to treat individual kinds of neoplasia.

By sharp contrast CK2 is constitutively active by itself, and no gain of function mutations of its catalytic (α and/or α’) subunits have been ever described which could account for malignant transformation. Nevertheless CK2 is almost invariably more elevated in tumour cells than it is in their “normal” counterparts, its over-expression potentiates the tumour phenotype induced by co-expression of oncogenes or by down-regulation of tumour suppressor genes^9^,^14^,^15^ and plenty of evidence is already available that CK2 is implicated in many of the cell biology phenomena associated with cancer^9^,^12^,^15^: it counteracts the efficacy of anticancer drugs, it strengthens the multi drug resistance (MDR) phenotype, it activates the chaperone machinery protecting the onco-kinome, and it sustains neo-vascularization. On top of this it plays a global role as an anti-apoptotic and pro-survival agent^4^,^16^. Consistent with this scenario CK2 inhibitors display a cytotoxic efficacy toward malignant cells of many different origins more pronounced than they do toward non-malignant cells^9^,^16^, an observation underlying the concept that cancer is “addicted” to high CK2 levels. A large repertoire of cell permeable CK2 inhibitors is already available^17^; most of these are ATP site directed with one, CX-4945, already in clinical trials for the treatment of cancer^18^,^19^.

The concept of “addiction” makes CK2 a multi-potential target as it implies that a wide repertoire of tumours generated by various genetic accidents display the common property of relying on a critical level of CK2 because it sustains and potentiates a number of signalling pathways which are specific to and/or hyper-activated in cancer cells whose survival relies on them. On the other hand the exploitation of CK2 as a valuable target in oncology has been hampered because, owing to its pleiotropicity and purported essential role, CK2 has been considered for a long time an “undruggable target”^20^. According to the concept of addiction, however, as opposed to that of “oncogenic determinism”, a partial down-regulation of the target may be as beneficial as its complete abrogation. On top of this, the concept that CK2 is “essential” needs to be revisited: although during embryogenesis this kinase is essential^21^,^22^ in adult cells its reduction may be not so critical.

To assess this crucial point we have now exploited the CRISPR/Cas9 technology to see if it is possible to generate viable clones lacking either the individual CK2 subunits or both its catalytic subunits. While in the case of the individual catalytic subunits this proved feasible with a variety of cells, including cancer cells (not shown), up to date the knockout of either both the catalytic subunits (α and α’) or the regulatory β subunit was only successful with the C2C12 myoblast cell line. Here we describe the properties of viable clones derived from C2C12 myoblasts entirely deprived of the whole CK2 catalytic activity (α/α’^(−/−)^). A proteomics analysis of these clones discloses substantial alterations that reflect their adaptation to live in the absence of CK2 activity and may shed light on cancer hallmarks that critically depend on CK2. These cells will also provide a valuable tool for discriminating between on- and off-target effects of currently used CK2 inhibitors and to explore the susceptibility of cells devoid of CK2 to undergo transformation upon transfection with oncogenes and with tumour suppressor genes.

## Results

### Validation of the CRISPR-based knockout technology to generate C2C12 cells devoid of CK2 subunits and catalytic activity

**Figure 1:**
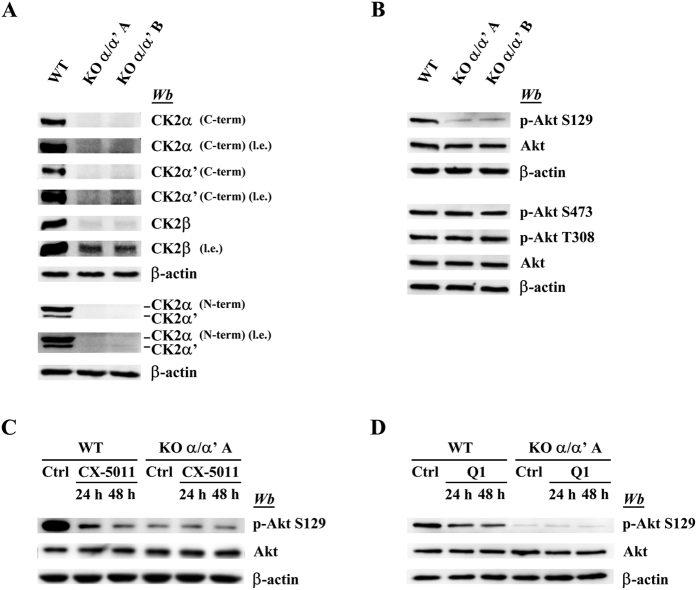
Characterization of CK2α/α’ knockout cells. Cellular lysates of wild type (WT) and of two different CK2α/α’ knockout clones (KO α/α’ A and KO α/α’ B) were analyzed by western blot with the indicated antibodies. (**A**) Analysis of the CK2 subunits (l.e.: long exposure). To detect the CK2 catalytic subunits two different antibodies that specifically recognize α or α’ (C-term) or an antibody able to recognize both catalytic subunits (N-term) were used. (**B**) Comparison of the Akt phosphorylation level between WT and knockout clones. (**C,D**) WT cells and knockout clones were treated with 2 μM CX-5011 (**C**) or with 20 μM Quinalizarin (Q1) for 24 and 48 h. Ctrl (control) cells refer to vehicle (DMSO) treated cells. Cell lysates were analyzed by western blotting with the indicated antibody. β-actin is the loading control.

Figure 1 shows that by exploiting the CRISPR/Cas9 genome editing technology (see Methods section) C2C12 cells (named clone A and clone B) were generated, where both the catalytic subunits of CK2 (α and α’) have disappeared. This is documented both by western blots of the individual subunits (Fig. 1A) and by monitoring the phosphorylation of Ser129 of Akt, notoriously affected by CK2^23^ (Fig. 1B). pS129 largely disappears in cells where both subunits are suppressed, at variance with the phosphorylation of Akt T308 and S473, which is not catalysed by CK2 and is consequently comparable in wild type (WT) and CK2 null cells (Fig. 1B). It should be also noted that the CK2 inhibitor CX-5011^24^ dramatically reduces the phosphorylation of S129 in WT cells, while having no effect on its residual phosphorylation occurring in the CK2α/α’^(−/−)^ cells (Fig. 1C). Same results have been obtained with the CK2 inhibitor Quinalizarin, structurally unrelated to CX-5011, confirming that the residual phosphorylation should be attributed to a different kinase (Fig. 1D).

**Figure 2:**
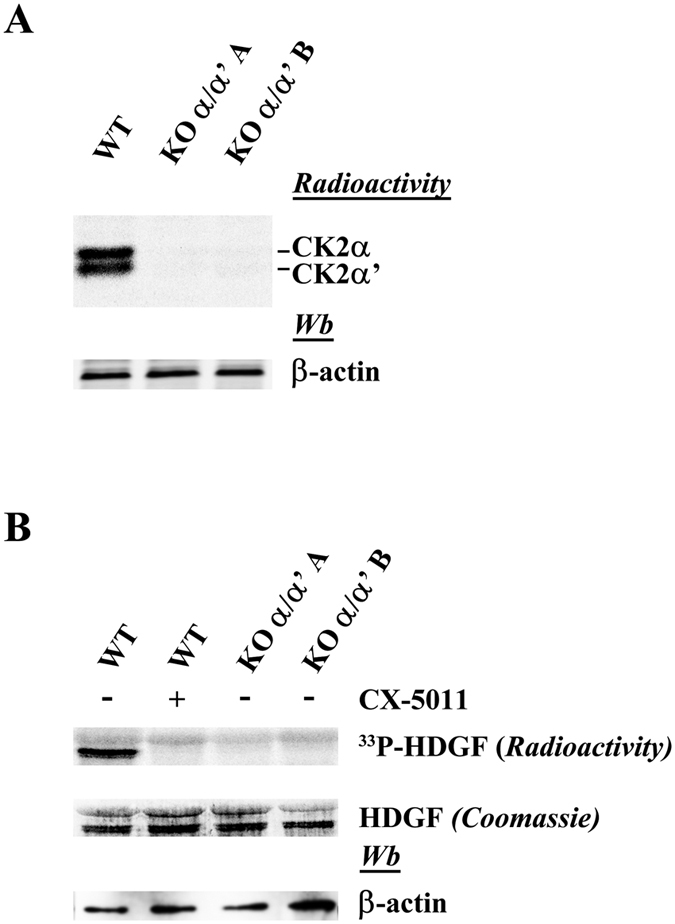
CK2 activity in CK2α/α’ knockout cells. CK2 activities were analyzed in WT and in CK2α/α’ knockout clones by in gel assay (**A**) and phosphorylation of exogenous substrate (GST-HDGF) in cell lysates (**B**). A 30 μg of lysate proteins were loaded on a polyacrylamide gel containing the CK2-substrate β-casein and CK2α/α’ activity was detected as detailed in Methods. (**B**) Cellular kinase activity of CK2 was tested *in vitro* in a phosphorylation medium containing lysate proteins, [γ^33^P]ATP and the protein substrate GST-HDGF as described in Methods. 100 nM CX-5011 was added where indicated.

The efficacy of knocking out CK2 catalytic subunits is confirmed by the experiments shown in Fig. 2, where CK2 activity of C2C12 cells is monitored with the aid of artificial substrates, either casein, by in-gel assay (A), or GST-HDGF, by *in vitro* assay (B). In both cases the double knockout of α/α’ promotes the disappearance of catalytic activity, which is entirely lost in the in-gel experiment confirming by another criterion the absence of even traces of the catalytic subunits, in accordance with the western blot analysis (see Fig. 1A). Likewise the phosphorylation of the exogenous recombinant protein GST-HDGF, previously described as a CK2 substrate^6^ becomes undetectable in CK2α/α’ knockout lysates (Fig. 2B). Finally to precisely determine the genotype and confirm that both alleles have been edited, Sanger sequencing was performed on PCR products amplified from genomic DNA spanning the crRNA target site (Figs S1 and S2). Clonal line A presents the same mutation in both alleles of *Csnk2a1* (CK2α) and *Csnk2a2* (CK2α’) genes (homozygous mutations) (Fig. S1), while clonal line B presents mutations but allelic events are unclear in both genes (heterozygous mutations) (Fig. S2). To confirm editing events on single alleles in clonal line B, PCR product has been cloned and sequenced. Sanger sequencing confirms two different mutations for both alleles in *Csnk2a1* and *Csnk2a2* genes of clonal line B (Fig. S2).

**Figure 3:**
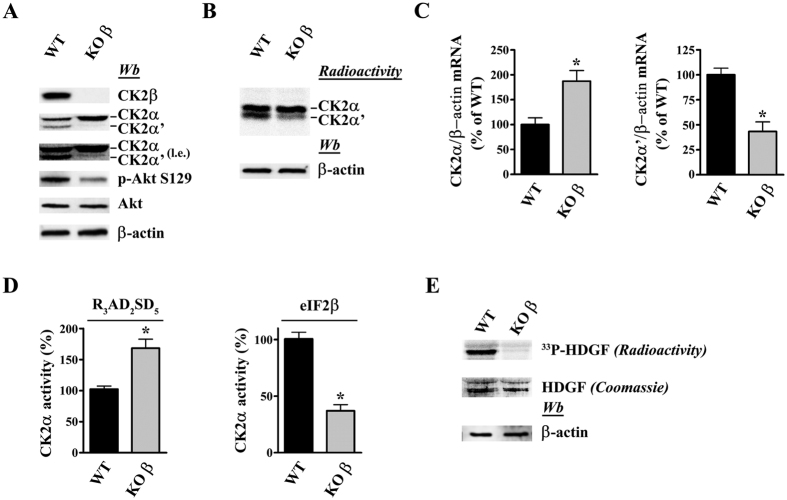
Characterization of CK2β knockout cells. (**A**) Cell lysates of WT and CK2β knockout clones were analyzed by western blot with the indicated antibodies. β-actin is the loading control. (**B**) In gel kinase assay. 30 μg of lysate proteins were loaded on a polyacrylamide gel containing the CK2-substrate β-casein and CK2α/α’ activity was detected as detailed in Methods. (**C**) Relative quantitation of gene expression (qRT-PCR) of CK2α and CK2α’ in WT and in KO β cells. The internal control was β-actin. Reported values are means ± SD of three independent experiments. Significance by Student’s t statistic: p-value ≤ 0.001 (*). (**D**) Cellular kinase activity of CK2 was tested *in vitro* in a phosphorylation medium containing lysate proteins, [γ^33^P]ATP and the peptide-substrates RRRADDSDDDDD or eIF2β[1-22] as detailed in Materials and Methods. Kinase activity is expressed as the percentage of the activity of the KO clones respect to the WT. Reported values are means ± SD of four independent experiments. Significance by Student’s t statistic: p-value ≤ 0.01 (*). (**E**) Cellular kinase activity of CK2 was tested *in vitro* in a phosphorylation medium containing lysate proteins, [γ^33^P]ATP and protein substrate GST-HDGF as described in Methods.

In Fig. 3 the effect of knocking out the regulatory β subunit of CK2 is examined. Sanger sequence analysis of the clonal line confirms the presence of an indel mutation in both alleles of *Csnk2b* (CK2β) gene (homozygous genotype) (Fig. S3). Two points deserve attention: firstly the lack of β substantially affects the level of the two catalytic subunits in opposite directions: the by far predominant α subunit significantly increases, while the α’ subunit becomes even fainter (Fig. 3A). In gel kinase assay confirms western blotting analysis (Fig. 3B). qRT-PCR analysis shows a change in mRNA expression of both CK2α and CK2α’ genes in KOβ cells, where the first is upregulated while the second is strongly reduced (Fig. 3C) as also observed at protein level. This finding, in agreement with the mutual transcriptional regulation of CK2 subunits as described by Pyerin group^25^, demonstrates that the alteration of the catalytic subunits of CK2 in the absence of β takes place at transcriptional level.

Secondly, as expected, the phosphorylation of two specific peptide substrates is affected by β in different manners (Fig. 3D), with the R_3_AD_2_SD_5_ peptide reflecting the overall increment of catalytic activity due to the α subunit (in accordance with previous observations in muscle-specific CK2β knockout mice^26^) while the eIF2β [1-22] peptide, whose phosphorylation is β dependent^27^, is phosphorylated by the lysates of β knockout cells much less readily than it is by the WT lysates. Similarly, the endogenous target Akt S129 (Fig. 3A) and the phosphorylation in CK2β knockout lysates of the recombinant protein GST-HDGF (Fig. 3E) resembles the eIF2β peptide in that their phosphorylations are largely dependent on availability of the β subunit, consistent with the concept that in living cells these targets are phosphorylated by the CK2 heterotetrameric holoenzyme rather than by the isolated catalytic subunits.

### Quantitative proteomics analysis of cells lacking CK2 catalytic subunits

For a deeper dissection of the biological functions of CK2 and to shed light on functional adaptations of CK2 null cells we have performed a quantitative proteomics analysis of CK2α/α’^(−/−)^ cells. Two distinct clones have been analysed in order to be able to compare the results not only with WT cells but also between the two clones.

**Figure 4:**
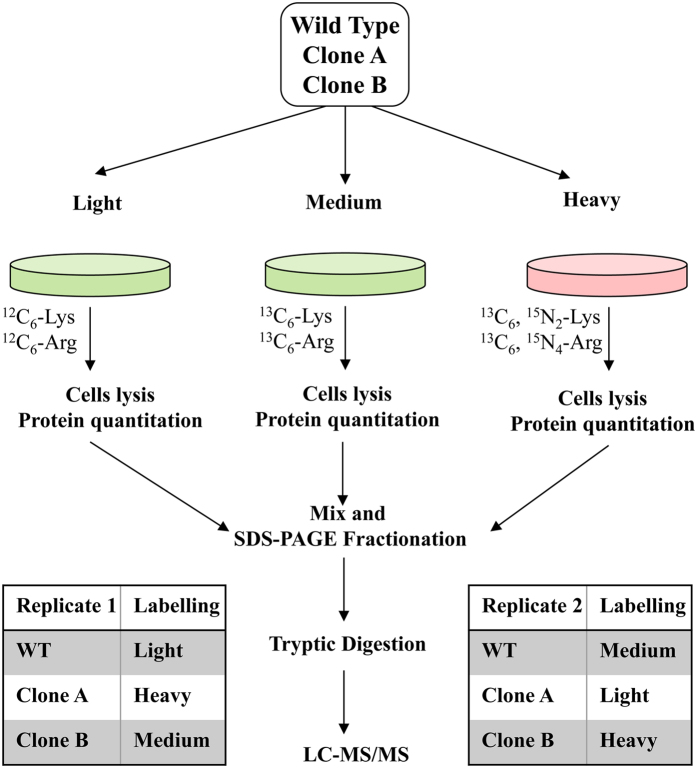
Workflow of SILAC Experiments. WT cells, clone **A** and clone **B** were labeled with stable isotopes of Lys and Arg using a label swapping strategy, as indicated. After 7 doublings, cells were lysed, protein quantified and mixed in a 1:1:1 ratio. Proteins were then separated by SDS-PAGE and in-gel digested with trypsin. Peptide mixtures were then finally analyzed my LC-MS/MS as described in Materials and Methods.

The quantitative proteomics experiment conducted using a SILAC approach was performed on two biological replicates and with a label-swapping strategy, as summarized in Fig. 4. For each of the replicates we compared the protein expression levels of C2C12 WT cells with those of the two clones lacking both catalytic subunits of CK2 (clone A and B). We focused our attention on proteins that were found to be significantly altered in both clones and in both replicates, so that the observed changes have the highest probability to be attributable to the lack of CK2α and α’ subunits, rather than to any side effect of the CRISPR/Cas9 procedure or to incomplete labelling and other experimental errors associated with the SILAC procedure^28^.

### Main outcomes of the proteomics analysis

Table 1 summarizes the main pieces of information provided by the MS analysis of C2C12 cells either WT or two clones in which the two catalytic subunits of CK2 have been suppressed by the CRISPR knockout methodology.

The number of proteins identified and quantified was 1618 and 1672 in replicate 1 and 2 respectively. 1416 proteins were quantified in both replicates and only these were considered for subsequent analyses.

Proteins whose level was significantly altered by at least 50% and that were statistically significant (p-value ≤ 0.05) with respect to WT were 301 and 319 in clone A and B, respectively. Of these, the proteins that showed an increased expression level (Fold Change ≥ 1.5) with respect to WT, were 186 and 132 for clone A and B respectively, while those decreased (Fold Change ≤ −1.5) were 115 and 187 for clone A and B respectively. The proteins that were significantly altered and in common to both clones were in total 240 (listed in Table S1), of which 126 showed a significant increased expression (p-value ≤ 0.05 and Fold Change ≥ 1.5) with respect to the WT and 114 were significantly decreased (p-value ≤ 0.05 and Fold Change ≤ −1.5).

As it is possible to observe in Figures S4 and S5, the logarithmic distribution of SILAC ratios highlights how both clones are characterized by a number of proteins that show significantly altered abundance with respect to the WT cells. What is worth mentioning is that there is also a significant difference between the two clones, as can be easily inferred from the shape of the green curve relative to the comparison between clone A and clone B (Fig. S4). This allows to conclude that clones A and B are characterized by two distinct proteomic profiles and for this reason we decided to consider in our downstream analyses only those proteins that were altered in the same way in both clones with respect to the WT cells.

Next we decided to assess the quality of the quantitative data across biological replicates. Figure S6 shows the distribution of log2 SILAC ratios for biological replicate 1 *vs* replicate 2 for the entire set of proteins for clones A and B (upper and lower panels, respectively). As it is possible to judge from the distribution of the values, both clones are characterized by a fair reproducibility of quantitative data, although clone B shows a higher degree of reproducibility across replicates. The observed discrepancies of SILAC quantitative data across biological replicates can be explained by a number of factors (incomplete labelling, different growth rates of cells in different medium, arginine-to-proline conversion, errors in the mixing of the samples, etc.). All these discrepancies can be efficiently taken care of by the application of the label-swapping strategy we have applied in our workflow^28^. For our downstream analyses therefore we only relied on those proteins that were altered with respect to the WT cells both in clone A and clone B and that showed a statistical significance (p-value ≤ 0.05 and with a Fold Change ≥ 1.5 or ≤ −1.5, as described in the Methods section).

### Validation of the proteomics data by immunoblot analysis of proteins whose expression is altered

**Figure 5:**
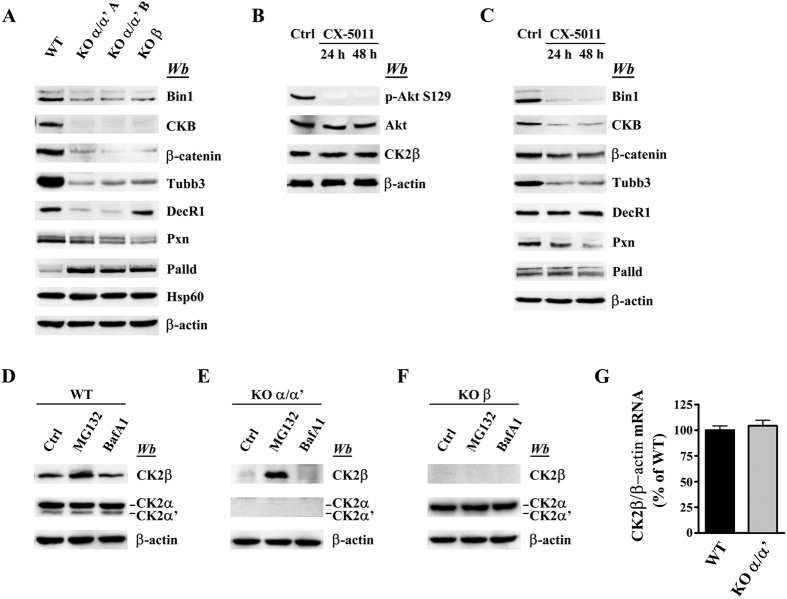
Validation of the proteomics analysis. (**A**) Cell lysates of WT, two different CK2α/α’ knockout clones (KO α/α’ A and KO α/α’ B), and CK2β knockout clone (KO β) were analyzed by western blot with the indicated antibodies. β-actin and Hsp60 are the loading controls. (**B,C**) WT cells were treated with 3 μM CX-5011 for 24 or 48 h. Ctrl (control) cells refer to vehicle (DMSO) treated cells. Cell lysates were analyzed by western blotting with the indicated antibodies. Inhibitor efficacy was verified assaying the phosphorylation of the CK2 specific site S129 on Akt (**B**). (**D,E,F**) WT, CK2α/α’, and CK2β knockout cells were treated for 6 h with DMSO (Ctrl), or with the proteasomal inhibitor MG132 (10 μM), or with the lysosomal inhibitor bafylomicin A1 (50 nM). Cell lysates were analyzed by western blotting with the indicated antibodies. β-actin is the loading control. (**G**) Relative quantitation of CK2β gene expression (qRT-PCR) in WT and KO α/α’ clone. The internal control was β-actin. No significative difference between WT and KO α/α’ clone was observed. Reported values are means ± SD of three independent experiments (significance by Student’s t statistic: p-value ≤ 0.05).

To validate the reliability of quantitative data provided by the SILAC approach we focused our attention on a subset of proteins, whose level is substantially altered (50% or more) in both α/α’^(−/−)^ clones and whose antibodies are available. These were: creatine kinase B (CKB), tubulin beta-3 chain (Tubb 3), β-catenin, Bin1, paxillin (Pxn), palladin (palld) and DecR1. As shown in Fig. 5A all these proteins also undergo a dramatic alteration in the α/α’^(−/−)^ cells if their level is evaluated by western blot analysis, in accordance with the SILAC data. The same applies, as already pointed out, to the β-subunit of CK2 itself (see Fig. 1A).

In order to check if and to what extent these alterations are directly attributable to the lack of CK2 catalytic activity, rather than, more in general, to the absence of CK2 subunits, advantage has been taken of CX-5011, a compound strictly related to the first-in-class CK2 inhibitor CX-4945, but even more selective than this^24^. As shown in Fig. 5C four proteins, Bin1, CKB, Tubb3 and Pxn, respond to CX-5011 treatment in the same manner as they do upon CK2α/α’ knockout, with a dramatic drop in their level. Such a parallelism however was not observed in the case of Palladin, whose expression is increased upon CK2 knocking out, while being unaffected or even slightly decreased by treating WT cells with CX-5011, and of the mitochondrial protein DecR1 which is unaffected by CX-5011 treatment (Fig. 5C) despite its decrement observed in CK2α/α’ KO cells (Fig. 5A). Likewise, β-catenin is reduced by CX-5011 treatment less than it is in the CK2 null cells. The same applies to the β-subunit of CK2, whose level is unaffected by CX-5011, being instead almost completely nullified in CK2α/α’^(−/−)^ cells (compare Fig. 5B to Fig. 1A). In this latter case the most likely explanation is that the isolated β subunit is unstable unless it associates with the catalytic subunits to give rise to CK2 holoenzyme: as already shown in fact^29^ β not assembled into the holoenzyme is rapidly degraded. Such an explanation is now confirmed by the observation that if cells are treated with the proteasome inhibitor MG132 (yet not with the lysosome inhibitor bafilomycin A1) β becomes detectable also in the CK2 null cells reaching a level comparable to that found in WT cells (compare Fig. 5D and E) while there is no difference in CK2β mRNA level between WT and CK2α/α’^(−/−)^ cells (Fig. 5G). Moreover CK2α/α’ subunits do not change in response to MG132 in both WT and KOβ cells confirming that the absence of CK2β does not affect the stability of the catalytic subunits in cells.

Intriguingly knocking out the non-catalytic β subunit alone, already shown to decrease the phosphorylation of Akt S129 (see above, Fig. 3A), also displays the same effect of knocking down both catalytic subunits on the level of most of the proteins considered in Fig. 5A. This might suggest that CK2 signalling in C2C12 cells is mainly performed by the holoenzyme, with an only marginal contribution of the isolated catalytic subunits. Such a conclusion however is challenged on one side by the observation that in at least one case, the mitochondrial protein DecR1, the effect of knocking out β does not mimic that of suppressing both catalytic subunits, on the other by the finding that, as shown above (Fig. 3) knocking out β promotes an overall increase of the activity of the catalytic subunits, rather than their disappearance, as one would expect if inside the cell they were functional only in combination with β, to generate the holoenzyme. The issue is further complicated by the observation that deletion of β has opposite effect on the level of the two catalytic subunits α and α’ (Fig. 3A) which have been shown to have different roles in CK2 driven processes^4^,^30^. For the time being therefore the role of CK2 catalytic subunits in CK2β null cells remains an open question.

### Subcellular localization and functional analysis of proteins whose level is significantly altered in CK2 null C2C12 cells

To gain information about which cellular processes are altered in cells where CK2 activity is suppressed, a number of bio-informatics tools have been exploited to perform a functional analysis of proteins whose level is significantly altered (+/−50%) in CK2α/α’^(−/−)^ cells.

**Figure 6:**
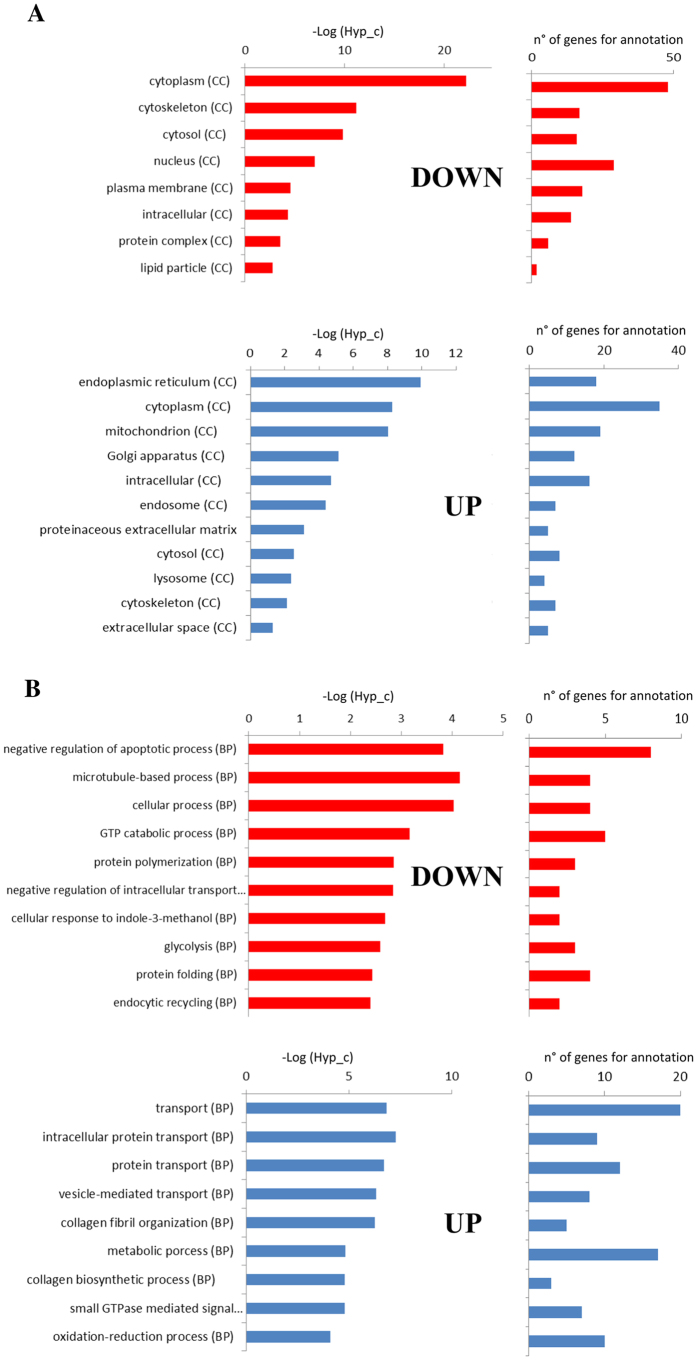
Functional analysis of proteins significantly altered (±50%) in CK2α/α’^(−/−)^ clones. Subcellular localization (**A**) and cell pathway analysis (**B**) of protein significantly altered (±50%) (downregulated in red and overexpressed in blue) in CK2α/α’^(−/−)^ clones assigned using GeneCoDis3 webserver^50^. Hyp_c: corrected value of hypergeometrical test. Only results with p-values ≤ 0.01 are shown.

We have separately analysed the two lists of up- and down-regulated proteins for their localization (Fig. 6A) and biological functions (Fig. 6B). It should be noted that up- and down-regulated proteins tend to segregate into distinct sub-cellular compartments: the former, e.g., are particularly represented in mitochondria, Golgi apparatus, and E.R., and are also found among extracellular proteins, where down-regulated proteins are nearly absent. In contrast these latter are numerous in nuclei and plasma membrane, where no appreciable presence of up-regulated proteins is detected. Moreover several cytoskeletal proteins are downregulated in KO clones (Fig. 6A), underscoring a key role of this kinase in regulating cytoskeletal structures and a potential involvement in all cytoskeleton based processes, as previously suggested^31^. The functional analysis shown in Fig. 6B also supports distinct roles for up-regulated proteins (largely implicated in transport, metabolic processes, redox reactions and collagen related functions) and down-regulated ones, a significant number of which are instead negative regulators of apoptosis, proteins involved in microtubule-based processes, glycolytic enzymes and molecules implicated in protein folding and polymerization.

Such a scenario is corroborated by the string analysis (Fig. S7). To note in particular the clusters of functionally interconnected up-regulated proteins (Fig. S7A) implicated in collagen metabolism/extracellular matrix functionality, in vesicular transport/secretory pathway and in mitochondrial oxidative phosphorylation. By contrast, and in agreement with the functional analysis of Fig. 6B, the tightest cluster of down-regulated proteins (Fig. S7B) pertains to glycolysis with other clusters composed of proteins implicated in epithelial-mesenchymal transition and cell migration.

To get a deeper insight into the physiological/pathological consequences expected upon alterations of proteins up and down regulated in C2C12 CK2 null cells a further analysis has been performed with the software Ingenuity Pathways Analysis. The results reported in Table S2, disclose that among the pathways which seem to be most affected by the lack of CK2, the following emerge: remodelling/signalling of epithelial adherens junctions, glycolysis and gluconeogenesis, citrulline biosynthesis, protein ubiquitination pathway, 14-3-3-mediated signalling, and integrin signalling.

For some of these, the involvement of CK2 does not come as a surprise, in particular for what concerns the role of CK2 in protein degradation through the ubiquitination pathway^32^,^33^,^34^.

The involvement of CK2 in the adherens and tight junction dynamics was also known^35^,^36^,^37^, however the very strong perturbations observed in these pathways by the loss of CK2α/α’ subunits seem to point to a crucial role played by this kinase in the adherens and tight junction regulation.

On the other hand, our result also suggest a strong involvement of CK2 in metabolism, with special reference to glycolytic process, an aspect which is worthy of further investigations.

**Figure 7:**
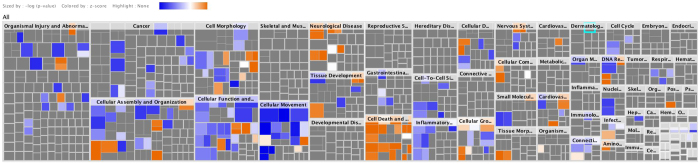
Ingenuity pathway analysis. The figure shows the impact of the lack of CK2 catalytic subunits on different diseases and biological functions. The color indicates activation (orange) or deactivation (blue) of the different functions belonging to the various categories.

A diseases & function analysis summarized in mosaic form (Fig. 7 and Table S3) provides some interesting hints. While a number of pathological situations (e.g. hereditary diseases, developmental disorders, and skeletal and muscle disorders) seem to be hardly affected by the proteomics alterations occurring in cells devoid of CK2, it appears that these alterations affect in opposite manners a number of functions that are relevant to other pathological situations. In particular the lack of CK2 predominantly inactivates processes relevant to cancer, to cellular movement, to cellular function and maintenance, to cell-to-cell signalling and interaction, and to inflammatory response. According to this analysis, the most deactivated processes (i.e. those with the most negative Z-score) are those involved in migration and invasion of cancer cells, a result that confirms the key role played by CK2 in tumour progression. By contrast, processes that are activated (positive Z-score) by the lack of CK2 are relevant to the categories of cell death and survival and neurological diseases, with special reference to the processes of protein ubiquitination, fragmentation of DNA, and anoikis. It may be interesting to note that the outcome of this analysis is in striking accordance with old observations concerning the up-regulation of CK2 expression in a variety of tumours as opposed to its downregulation in Alzheimer brain (reviewed by^38^).

## Discussion

To the best of our knowledge the data presented provide the first example of viable cells in which both the catalytic subunits of CK2, and therefore the whole enzymatic activity of this pleiotropic kinase, have been suppressed. Interestingly knocking out both the catalytic subunits of CK2 also promotes an almost complete disappearance of the non-catalytic β-subunit, which is accounted for by the prompt proteasomal degradation of free β, since in the presence of proteasome inhibitors β accumulates in α/α’^(−/−)^ cells and in wild type cells to similar extents. By contrast knocking out β promotes an overall increase of CK2 catalytic activity, due to a substantial increase of the by far predominant α subunit. These observations are consistent with a scenario whereby the catalytic and the regulatory subunits of CK2 may also play separate functions, independent of each other, in addition to the well-established role of giving rise to the heterotetrameric CK2 holoenzyme. The crucial role of this latter as a generator of CK2 signalling, anyway, is underscored by the observation that many of the effects promoted by knocking out the two catalytic subunits are also occurring upon knocking out β alone. This applies, e.g. to the drop in phosphorylation of Akt Ser-129 and to the altered expression of several proteins listed in Fig. 5. A notable exception however, is provided by the different response of the mitochondrial protein DecR1, suggesting that the isolated catalytic subunits may play also roles distinct from those of the holoenzyme. A thorough proteomics analysis of cells deprived of the β subunit will be needed to clarify this aspect.

Regardless to the rapid degradation of the regulatory β-subunit in our CK2α/α’^(−/−)^ cells the evidence that these clones are entirely devoid of CK2 catalytic activity is solid and incontrovertible, being grounded on the total absence of both the proteins and their enzymatic activity (see Figs 1 and 2). It should be noted in this respect that the weak residual phosphorylation of Akt Ser-129 in the CK2 KO cells is not attributable to CK2 as it is unaffected by two structurally unrelated CK2 inhibitors CX-5011 and Quinalizarin (see Fig. 1C). Finally, genetic analysis of these KO clones confirms that both alleles of CK2α and CK2α’ genes have been edited (see Figs S1 and S2). These cells therefore provide a valuable tool to analyse proteomics alterations caused by the absence of this highly pleiotropic kinase and reflecting in functional rearrangements.

Several reports have already been published dealing with (phospho)proteomic alterations promoted by treating cells with CK2 inhibitors (e.g. refs 39, 40, 41, 42). It should be underlined however that those conditions are different from cell clones viable in the absence of CK2 catalytic subunits. Firstly in fact the selectivity of kinase inhibitors, even the most specific ones, is never absolute and side effects are unavoidable, as also shown in the case of selective CK2 inhibitors^43^. Secondly, suppression of catalytic activity does not necessarily prevent the interaction of CK2 with the plethora of protein partners other than its substrates^44^; thirdly and most important, the abrupt and transient pharmacological down-regulation of CK2 generates a scenario whereby the multifarious targets of this pleiotropic kinase lose their phosphates gradually and at variable rates^39^,^40^, cooperating to a cellular stress ending up with apoptosis, a typical outcome of CK2 inhibition^16^. By sharp contrast our clones are composed of cells which have succeeded to escape apoptosis and are viable despite being entirely devoid of CK2 catalytic subunits from the very beginning. In these cells the plethora of physiological CK2 targets have never got the opportunity of becoming phosphorylated by this enzyme.

The quantitative proteomics analysis presented here suggests that viability of C2C12 cells in the absence of CK2 is accompanied by proteomics alterations consistent with a functional and metabolic rewiring. Specifically, downregulated proteins belong to the following categories: (i) cytoskeletal proteins denoting a primary role of CK2 in processes such as cell migration, epithelial-mesenchimal transition, remodelling/signalling of epithelial adherens junctions, and in general in all processes where cytoskeletal components are primary involved; (ii) negative regulator of apoptosis, confirming the well-known role of CK2 in antiapoptotic process; (iii) glycolytic enzymes, suggesting a unanticipated role of CK2 in metabolic processes. The upregulated proteins instead would indicate a negative role of CK2 in processes implicated in collagen metabolism/extracellular matrix functionality, in vesicular transport/secretory pathway and in mitochondrial bioenergetics functions. The opposite role in the glycolytic pathways and in mitochondrial bioenergetics would also fit in very well with the concept of addiction, grounded on the assumption that elevated CK2 generates a cellular environment especially favourable to neoplasia. Consistently, a diseases and function analysis performed with the Ingenuity Pathways tool reveals that the process most deactivated in the absence of CK2 are those involved in migration and invasion of cancer cells. On the other hand, absence of CK2 appears to have a potential favourable effect on neurological diseases.

## Methods

### Materials

[γ-^33^P]ATP was purchased from Hartmann Analytic GmbH (Braunschweig Germany). Protease inhibitor cocktail was from Calbiochem (Darmstadt, Germany), while phosphatase inhibitor cocktails 2 and 3 were from Sigma-Aldrich (Dorset, UK). CX-5011 (5-(3-ethynylphenylamino)pyrimido[4,5-c]quinoline-8-carboxylic acid) was purchased from Glixx Laboratories (South Borough, MA). Quinalizarin was purchased from ACP Chemicals Inc (Montreal, Canada). Solutions were made in dimethylsulfoxide (DMSO). Peptides RRRADDSDDDDD and MSGDEMIFDPTMSKKKKKKKKP (eIF2β[1-22]) were kindly provided by Prof. Oriano Marin (University of Padova). gRNAs designs and reagents for genome editing of CK2α and CK2α’ genes were supplied by Horizon Discovery (www.horizondiscovery.com), for genome editing of CK2β were purchased from DNA 2.0, Inc. Antiserum against CK2α subunit was raised in rabbit using the peptide reproducing the sequence of the human catalytic subunit at the C-terminus (376–391). Anti CK2β antibody was from Abcam. Anti Akt1, anti p-Akt S473 and p-Akt T308 antibodies were from Cell Signaling Technology (Danvers, MA), anti p-Akt1 S129 was from Abcam (Cambridge, UK), while anti-β-actin was purchased from Sigma-Aldrich. Anti CDC37 and anti CK2α’ were from Santa Cruz and anti p-CDC37 S13 antibodies were from Abcam. Anti paxillin, Bin 1, creatine kinase B, Tubulin beta-3 chain, and DecR1 antibodies were from GeneTex. Secondary antibodies towards rabbit and mouse IgG, conjugated to horse radish peroxidase, were from PerkinElmer. Labeled amino acids for SILAC experiments were purchased from Silantes GmbH (Muchen, Germany). Not labeled amino acids (L-arginine, L-lysine, L-glutamine and L-proline) were purchased from Sigma.

### Cell culture

C2C12 cells were maintained in 5% CO_2_ in DMEM supplemented with 10% FBS, 2 mM L-glutamine, 100 U/ml penicillin and 100 mM streptomycin, in an atmosphere containing 5% CO_2_.

### Cell lysis and western blotting analysis

Cells were detached, centrifuged, extensively washed with PBS and lysed by the addition of ice-cold buffer containing 20 mM Tris–HCl (pH 8.0), 150 mM NaCl, 2 mM EDTA, 2 mM EGTA, 1% Triton X-100 (v/v), protease inhibitor cocktail Complete (Roche) and phosphatase inhibitor Cocktail 2 and 3 (Sigma). After 20 min incubation on ice, the lysate was centrifuged 10 min at 10,000 × *g* at 4 °C. The supernatant was collected and protein concentration was determined by the Bradford method. Equal amounts of protein were loaded on 12% SDS-PAGE, blotted on Immobilon-P membranes (Millipore), processed by western blot with the indicated antibody, and detected by chemiluminescence on a Kodak Image Station 440MM PRO. Quantitation of the signal was obtained by analysis with the Kodak 1D Image software.

### CRISPR/Cas9-mediated genome editing

All-in-one plasmids expressing Cas9-DasherGFP and the sgRNA guide (pD1301-AD: CMV-Cas9-2A-GFP, Cas9-ElecD) to target the specific CK2 subunits were used. The sgRNA guide sequence are 5′-CATAATGTCATGATTGATCA-3′ (CK2α), 5′-GTTCACCTCGGCGTAGACCC-3′ (CK2α′), and 5′-TCCTGGTTCTGTGGGCTCCG-3′ (CK2β). Sequence for CK2β have been chosen using the CRISPR MultiTargeter tool^45^ and off targets have been excluded using GT-Scan tools^46^.

C2C12 cells were cultured in six-well dishes to 70–80% confluence. Cells were co-transfected with 1 μg of sgRNA plasmid and Lipofectamine 3000 according to manufacturing instructions. Forty-eight hours post-transfection, cells were pelleted in PBS and sorted in 96-well plates using fluorescence-activated cell sorting (FACS) with a FACSAria II cell sorter (BD BioSciences). Single cells were expanded to obtain individual clones. Individual clones were lysed and quantified as described above. The absence of the specific CK2 subunits was verified by western blotting, kinase activity assay and sanger sequence analysis. Sanger sequencing was performed on PCR products amplified from genomic DNA spanning the crispr RNA target. For this purpose genomic DNA of clonal cell lines was extracted using the genomic DNA purification kit (Thermo Fisher). The obtained DNA, after spectrophotometric quantification was amplified by PCR using iProof High-Fidelity Master Mix (Bio-Rad). For the amplification reaction we used the following primers pairs: the forward 5′-CCTGATTCCCTGGATTGTTG-3′ and reverse 5′-CAGGATGGTTCAGCTGGTTT-3′ primers to amplified the KO CK2α sequence;

The forward 5′-CGCTCCTCCTCTTGCTTG-3′ and reverse 5′-ACCCATAGGAAGCCCAAAGT-3′ primers to amplified the KO CK2α’ sequence;

The forward 5′-GCTTGGAGATGCTTCAGAGG-3′ and reverse 5′-GGCTTTGCACATTACCCAAC-3′ primers to amplified the KO CK2β sequence.

PCR products were separated by agarose gel electrophoresis, and appropriate bands were cut-out and sequenced to determine the genotype and whether one or both alleles had been mutated. For clonal lines presenting heterozygous genotype where allelic events were unclear the same PCR products were cloned in pGEM-T-Easy vector and 10–12 single bacterial colonies were sequenced and analyzed.

### qRT-PCR

The total RNA of duplicate samples of C2C12 myoblast cells (wild type, and knockout clonal lines) were extracted using the total RNA Purification Kit, (Norgen Biotek Corp.) and the RNA obtained was retrotranscribed using the SuperScript^®^ III Reverse Transcriptase (Thermo Fisher). RNA yield, were determined at the spectrophotometer and the cDNA of each sample was analyzed in triplicate to evaluate the gene expression in qRT-PCR using the SensiFAST SYBR No-ROX Kit (Bioline Reagents Ltd).

We used the following pairs of primers to analyze the gene expression. CK2α: forward 5′-AAGACCCTGTGTCACGAACC-3′ and reverse 5′-CTCTTGGCCTGGATGGTAAA-3′. CK2α’: forward 5′-CATGCACAGGGATGTGAAAC-3′ and reverse 5′-AGAATGGCTCCTTTCGGAAT-3′. CK2β: forward 5′-ATCGAACAGGCAGCTGAGAT-3′ and reverse 5′- GTGGTGGTGTCTGGAGGACT-3′.

The real-time PCR results are normalized against the control β-actin housekeeping genes using the primers forward 5′-GTCCCTCACCCTCCCAAAAG-3′ and reverse 5′-GCTGCCTCAACACCTCAACCC-3′.

The real-time PCR results are normalized against the control β-actin housekeeping gene.

All the amplification reactions were performed using the Rotor Gene 3000 instrument.

### CK2 kinase activity assay

Proteins from cell lysates (1 μg) were incubated for 10 min at 30 °C in 25 μl of a phosphorylation medium containing 50 mM Tris–HCl (pH 7.5), 100 mM NaCl, 10 mM MgCl_2_, 400 μM synthetic peptide-substrate RRRADDSDDDDD or MSGDEMIFDPTMSKKKKKKKKP (eIF2β) and 50 μM [γ-^33^P]ATP (1000 cpm/pmol). Assays were stopped by absorption onto phosphocellulose filters. Filters were washed four times in 75 mM phosphoric acid and analyzed by a Scintillation Counter (PerkinElmer).

### In-gel kinase assay of CK2α/α’

The activity displayed by CK2 subunits were determined by running cell lysates on an 11% SDS-PAGE containing the CK2-substrate β-casein (0.5 mg/ml). After electrophoresis, the activity of CK2α toward the co-localized β-casein was detected by incubating the gel with the above described phosphorylation medium containing 10 μM [γ^33^P]ATP^47^. Radioactive ^33^P-β-casein was evidenced by analyzing the dried gel with a Cyclone Plus Storage PhosphorSystem (PerkinElmer).

### Stable isotope labeling and cell lysis

Cells were grown in DMEM containing either unlabeled l-arginine and l-lysine (Arg^0^, Lys^0^) (light) or equimolar amounts of l-[^13^C_6_]-arginine and l-[^13^C_6_]-lysine (Arg^6^, Lys^6^) (medium) or l-[^13^C_6_,^15^N_4_]-arginine and l-[^13^C_6_,^15^N_2_]-lysine (Arg^10^, Lys^8^) (heavy) supplemented with 200 mg/L light proline to prevent the conversion of arginine to proline^48^, 2 mM L-glutamine, 1% penicillin/streptomycin, and 10% dialyzed Fetal Bovine Serum (Silantes GmbH). Cells were grown in SILAC medium for seven cell doublings. Labeled cells were solubilized by the addition of ice-cold buffer containing 20 mM HEPES (pH 8.0), 9 M urea, protease inhibitors EDTA-free (1 tablet/10 ml, Roche) and phosphatases inhibitors Cocktail 2 and 3 (Sigma) and sonicated. Cell debris was removed by centrifugation and SILAC-encoded samples were pooled at a ratio of 1:1:1.

### SDS-PAGE and in-gel protein digestion

For the SILAC experiment, 25 μg of total protein extracts from each of the 3 samples (WT, clone A and clone B) were mixed using a swapping strategy, as specified in Fig. 4. Proteins were loaded into a precast 4–12% Bis-Tris gel (NuPAGE, Thermo Fisher Scientific) and separated for 1 h at 25 mA. The gel was then stained with SimplyBlue SafeStain colloidal Coomassie (Invitrogen) and destained overnight in water. The 2 gel lanes relative to the 2 biological replicates were cut in 8 slices, each individually subjected to in-gel digestion with sequencing grade modified trypsin (Promega) as described elsewhere^49^. Peptides were extracted from the gel by 4 changes (20 min each) of 50% acetonitrile/0.1% formic acid and dried under vacuum.

### LC–MS/MS and data analysis

Samples were dissolved in 40 μL of 3% acetonitrile (ACN)/0.1% formic acid (FA) and analyzed by LC-MS/MS using a LTQ-Orbitrap XL mass spectrometer (Thermo Fisher Scientific) interfaced to a nano-HPLC Ultimate 3000 (Dionex - Thermo Fisher Scientific) as described in^40^. Peptides were loaded onto a trap column (C_18_, 300 μm I.D., 10 mm, Protecol, Analytical Technology) at a flow rate of 8 μL/min and then separated using a pico-frit column (75 μm I.D., 15 μM tip, 11 cm, New Objective) packed in-house with C_18_ material (Aeris Peptide 3.6 μm XB-C18, Phenomenex). The separation was achieved using 0.1% FA (Eluent A) and ACN/0.1% FA (eluent B) as mobile phases with a linear gradient of ACN from 3 to 50% in 90 min at a flow rate of 250 nL/min. Spray voltage was set to 1.2–1.3 kV with an ion source capillary temperature of 200 °C. The instrument was operating in data-dependent mode with a top-four acquisition method (a full scan at 60,000 resolution on the Orbitrap followed by the MS/MS fragmentation in the linear trap of the four most intense ions). To increase the number of identified proteins and the robustness of quantitative data, each sample was analyzed a first time, data obtained were searched with Mascot search engine (details are reported in the next paragraph) and an exclusion list was created with Proteome Discoverer software, based on all peptides that were positively identified. Each sample was then analyzed again under identical chromatographic and instrumental conditions, but for the application of the static exclusion list.

### Data Analysis

Raw files were analyzed with the software package Proteome Discoverer 1.4 (Thermo Fisher Scientific) connected to a Mascot search engine (version 2.2.4, Matrix Science,). Data were searched against the mouse section of the Uniprot database (version 2015.04.01; 53301 sequences). Enzyme specificity was set to trypsin with up to 1 missed cleavage. Mass tolerance was set to 10 ppm and to 0.8 Da for parent mass and fragment ions respectively.

Carbamidomethylation of cysteines was set as fixed modification, while the following variable modifications were used: [^13^C_6_]-Lys, [^13^C_6_]-Arg, [^13^C_6_,^15^N_2_]-Lys,[^13^C_6_,^15^N_4_]-Arg, and methionine oxidation. The reliability of peptide identification was assessed using the algorithm Percolator and a search against a randomized database, keeping into account only peptides identified with a q-value < 0.01 (99% confidence) and rank 1. Proteins were grouped into protein families, based on the principle of maximum parsimony and were considered as positively identified if at least 2 unique peptides per protein were identified with high confidence. Quantification of SILAC triplets was performed directly by Proteome Discoverer software, with the limitation of using only unique peptides for quantification purposes. Data obtained from both biological replicates were analyzed with a MudPIT protocol: all data obtained from the technical replicates (without and with the application of the exclusion list) were merged to produce a single output file. Only proteins quantified in both biological replicates were retained for further analysis. A two-tailed Z test was performed to select only proteins that showed a significant (p-value ≤ 0.05) increased/decreased expression. A further biological cut-off was applied by selecting only proteins with a fold change of at least 1.5 in both clones with respect to the WT cells.

### Bioinformatic analysis

Bioinformatic analyses to define cellular localization and protein functions were performed with Genecodis^50^ and STRING^51^. Ingenuity Pathways Analysis (Qiagen) was used to highlight pathways, networks, and diseases/biological functions particularly affected by the lack of the catalytic subunits of CK2.

## Additional Information

**How to cite this article**: Borgo, C. *et al*. Generation and quantitative proteomics analysis of CK2α/α’^(−/−)^ cells. *Sci. Rep.* **7**, 42409; doi: 10.1038/srep42409 (2017).

**Publisher's note:** Springer Nature remains neutral with regard to jurisdictional claims in published maps and institutional affiliations.

## Supplementary Material

Supplementary Figures and Table

Supplementary Table S1

Supplementary Table S2

Supplementary Table S3

## Figures and Tables

**Table 1:** SILAC proteomics analysis of WT and CK2α/α’^(−/−)^ cells.

	Replicate 1	Replicate 2
Peptide Spectrum Matches (PSM)	50227	47819
Unique peptides	15237	15856
Protein groups	1618	1672
Protein groups common to both replicates	1416	
Altered proteins clone A vs WT	301	
Altered proteins clone A vs WT (Fold Change ≥ 1.5)	186	
Altered proteins clone A vs WT (Fold Change ≤ −1.5)	115	
Altered proteins clone B vs WT	319	
Altered proteins clone B vs WT (Fold Change ≥ 1.5)	132	
Altered proteins clone B vs WT (Fold Change ≤ −1.5)	187	
Altered proteins common to clone A and B	240	
Altered proteins common to clone A and B (Fold Change ≥ 1.5)	126	
Altered proteins common to clone A and B (Fold Change ≤ −1.5)	114	

## References

[b1] Allende J. E. & Allende C. C. Protein kinases. 4. Protein kinase CK2: an enzyme with multiple substrates and a puzzling regulation. FASEB J 9, 313–323 (1995). 7896000 10.1096/fasebj.9.5.7896000

[b2] Pinna L. A. Protein kinase CK2: a challenge to canons. J Cell Sci 115, 3873–3878 (2002). 12244125 10.1242/jcs.00074

[b3] Meggio F. & Pinna L. A. One-thousand-and-one substrates of protein kinase CK2? FASEB J 17, 349–368 (2003). 12631575 10.1096/fj.02-0473rev

[b4] Litchfield D. W. Protein kinase CK2: structure, regulation and role in cellular decisions of life and death. Biochem J 369, 1–15 (2003). 12396231 10.1042/BJ20021469 PMC1223072

[b5] Allende C. C. & Allende J. E. Promiscuous subunit interactions: a possible mechanism for the regulation of protein kinase CK2. J Cell Biochem Suppl 30–31, 129–136 (1998). 9893264

[b6] Salvi M., Sarno S., Cesaro L., Nakamura H. & Pinna L. A. Extraordinary pleiotropy of protein kinase CK2 revealed by weblogo phosphoproteome analysis. Biochim Biophys Acta 1793, 847–859 (2009). 19339213 10.1016/j.bbamcr.2009.01.013

[b7] Cesaro L. & Salvi M. CK2 contribution to the generation of the human phosphoproteome in Protein kinase CK2- The Wiley-IUBMB Series on Biochemistry and Molecular Biology (ed. Pinna L. A.) 117–128. (Wiley, 2013).

[b8] Pinna L. A. The raison d’etre of constitutively active protein kinases: the lesson of CK2. Acc Chem Res 36, 378–384 (2003). 12809523 10.1021/ar020164f

[b9] Ruzzene M. & Pinna L. A. Addiction to protein kinase CK2: a common denominator of diverse cancer cells? Biochim Biophys Acta 1804, 499–504 (2010). 19665589 10.1016/j.bbapap.2009.07.018

[b10] St-Denis N. A. & Litchfield D. W. Protein kinase CK2 in health and disease: From birth to death: the role of protein kinase CK2 in the regulation of cell proliferation and survival. Cell Mol Life Sci 66, 1817–1829 (2009). 19387552 10.1007/s00018-009-9150-2 PMC11115660

[b11] Pinna L. A. & Allende J. E. Protein kinase CK2 in health and disease: Protein kinase CK2: an ugly duckling in the kinome pond. Cell Mol Life Sci 66, 1795–1799 (2009). 19387554 10.1007/s00018-009-9148-9 PMC11115792

[b12] Trembley J. H., Wang G., Unger G., Slaton J. & Ahmed K. Protein kinase CK2 in health and disease: CK2: a key player in cancer biology. Cell Mol Life Sci 66, 1858–1867 (2009). 19387548 10.1007/s00018-009-9154-y PMC4385580

[b13] Guerra B. & Issinger O. G. Protein kinase CK2 in human diseases. Curr Med Chem 15, 1870–1886 (2008). 18691045 10.2174/092986708785132933

[b14] Ortega C. E., Seidner Y. & Dominguez I. Mining CK2 in cancer. PLoS One 9, e115609 (2014). 25541719 10.1371/journal.pone.0115609 PMC4277308

[b15] Venerando A., Ruzzene M. & Pinna L. A. Casein kinase: the triple meaning of a misnomer. Biochem J 460, 141–156 (2014). 24825444 10.1042/BJ20140178

[b16] Ahmad K. A., Wang G., Unger G., Slaton J. & Ahmed K. Protein kinase CK2–a key suppressor of apoptosis. Adv Enzyme Regul 48, 179–187 (2008). 18492491 10.1016/j.advenzreg.2008.04.002 PMC2593134

[b17] Cozza G. & Pinna L. A. Casein kinases as potential therapeutic targets. Expert Opin Ther Targets 20, 319–340 (2016). 26565594 10.1517/14728222.2016.1091883

[b18] Martins L. R. . Activity of the clinical-stage CK2-specific inhibitor CX-4945 against chronic lymphocytic leukemia. Leukemia 28, 179–182 (2014). 23925046 10.1038/leu.2013.232

[b19] Pierre F. . Discovery and SAR of 5-(3-chlorophenylamino)benzo[c][2,6]naphthyridine-8-carboxylic acid (CX-4945), the first clinical stage inhibitor of protein kinase CK2 for the treatment of cancer. J Med Chem 54, 635–654 (2011). 21174434 10.1021/jm101251q

[b20] Cozza G., Bortolato A. & Moro S. How druggable is protein kinase CK2? Med Res Rev 30, 419–462 (2010). 19526464 10.1002/med.20164

[b21] Dominguez I. . CK2alpha is essential for embryonic morphogenesis. Mol Cell Biochem 356, 209–216 (2011). 21761203 10.1007/s11010-011-0961-8 PMC3756899

[b22] Lou D. Y. . The alpha catalytic subunit of protein kinase CK2 is required for mouse embryonic development. Mol Cell Biol 28, 131–139 (2008). 17954558 10.1128/MCB.01119-07 PMC2223292

[b23] Di Maira G. . Protein kinase CK2 phosphorylates and upregulates Akt/PKB. Cell Death Differ 12, 668–677 (2005). 15818404 10.1038/sj.cdd.4401604

[b24] Battistutta R. . Unprecedented selectivity and structural determinants of a new class of protein kinase CK2 inhibitors in clinical trials for the treatment of cancer. Biochemistry 50, 8478–8488 (2011). 21870818 10.1021/bi2008382

[b25] Pyerin W. & Ackermann K. The genes encoding human protein kinase CK2 and their functional links. Prog Nucleic Acid Res Mol Biol 74, 239–273 (2003). 14510078 10.1016/s0079-6603(03)01015-8

[b26] Cheusova T. . Casein kinase 2-dependent serine phosphorylation of MuSK regulates acetylcholine receptor aggregation at the neuromuscular junction. Genes Dev 20, 1800–1816 (2006). 16818610 10.1101/gad.375206 PMC1522076

[b27] Salvi M. . Discrimination between the activity of protein kinase CK2 holoenzyme and its catalytic subunits. FEBS Lett 580, 3948–3952 (2006). 16806200 10.1016/j.febslet.2006.06.031

[b28] Park S. S. . Effective correction of experimental errors in quantitative proteomics using stable isotope labeling by amino acids in cell culture (SILAC). J Proteomics 75, 3720–3732 (2012). 22575385 10.1016/j.jprot.2012.04.035 PMC3394155

[b29] Luscher B. & Litchfield D. W. Biosynthesis of casein kinase II in lymphoid cell lines. Eur J Biochem 220, 521–526 (1994). 8125110 10.1111/j.1432-1033.1994.tb18651.x

[b30] Turowec J. P., Vilk G., Gabriel M. & Litchfield D. W. Characterizing the convergence of protein kinase CK2 and caspase-3 reveals isoform-specific phosphorylation of caspase-3 by CK2alpha’: implications for pathological roles of CK2 in promoting cancer cell survival. Oncotarget 4, 560–571 (2013). 23599180 10.18632/oncotarget.948 PMC3720604

[b31] Canton D. A. & Litchfield D. W. The shape of things to come: an emerging role for protein kinase CK2 in the regulation of cell morphology and the cytoskeleton. Cell Signal 18, 267–275 (2006). 16126370 10.1016/j.cellsig.2005.07.008

[b32] Semplici F., Meggio F., Pinna L. A. & Oliviero S. CK2-dependent phosphorylation of the E2 ubiquitin conjugating enzyme UBC3B induces its interaction with beta-TrCP and enhances beta-catenin degradation. Oncogene 21, 3978–3987 (2002). 12037680 10.1038/sj.onc.1205574

[b33] Scaglioni P. P. . A CK2-dependent mechanism for degradation of the PML tumor suppressor. Cell 126, 269–283 (2006). 16873060 10.1016/j.cell.2006.05.041

[b34] Tsuchiya Y. . The casein kinase 2-nrf1 axis controls the clearance of ubiquitinated proteins by regulating proteasome gene expression. Mol Cell Biol 33, 3461–3472 (2013). 23816881 10.1128/MCB.01271-12 PMC3753846

[b35] Lee N. P. & Cheng C. Y. Protein kinases and adherens junction dynamics in the seminiferous epithelium of the rat testis. J Cell Physiol 202, 344–360 (2005). 15389520 10.1002/jcp.20119

[b36] Serres M. . The disruption of adherens junctions is associated with a decrease of E-cadherin phosphorylation by protein kinase CK2. Exp Cell Res 257, 255–264 (2000). 10837139 10.1006/excr.2000.4895

[b37] Dorfel M. J. . CK2-dependent phosphorylation of occludin regulates the interaction with ZO-proteins and tight junction integrity. Cell Commun Signal 11, 40 (2013). 23758859 10.1186/1478-811X-11-40 PMC3695765

[b38] Issinger O. G. Casein kinases: pleiotropic mediators of cellular regulation. Pharmacol Ther 59, 1–30 (1993). 8259381 10.1016/0163-7258(93)90039-g

[b39] Franchin C. . Quantitative analysis of a phosphoproteome readily altered by the protein kinase CK2 inhibitor quinalizarin in HEK-293T cells. Biochim Biophys Acta 1854, 609–623 (2015). 25278378 10.1016/j.bbapap.2014.09.017

[b40] Franchin C., Salvi M., Arrigoni G. & Pinna L. A. Proteomics perturbations promoted by the protein kinase CK2 inhibitor quinalizarin. Biochim Biophys Acta 1854, 1676–1686 (2015). 25882195 10.1016/j.bbapap.2015.04.002

[b41] Le Bihan T. . Label-free quantitative analysis of the casein kinase 2-responsive phosphoproteome of the marine minimal model species Ostreococcus tauri. Proteomics 15, 4135–4144 (2015). 25930153 10.1002/pmic.201500086 PMC4716292

[b42] Rodriguez-Ulloa A. . Proteomic profile regulated by the anticancer peptide CIGB-300 in non-small cell lung cancer (NSCLC) cells. J Proteome Res 9, 5473–5483 (2010). 20804217 10.1021/pr100728v

[b43] Kim H. . Identification of a novel function of CX-4945 as a splicing regulator. PLoS One 9, e94978 (2014). 24743259 10.1371/journal.pone.0094978 PMC3990583

[b44] Montenarh M. G. C. The Interactome of protein kinase CK2. (The Wiley-IUBMB Series on Biochemistry and Molecular Biology, 2013).

[b45] Prykhozhij S. V., Rajan V., Gaston D. & Berman J. N. CRISPR multitargeter: a web tool to find common and unique CRISPR single guide RNA targets in a set of similar sequences. PLoS One 10, e0119372 (2015). 25742428 10.1371/journal.pone.0119372 PMC4351176

[b46] O’Brien A. & Bailey T. L. GT-Scan: identifying unique genomic targets. Bioinformatics 30, 2673–2675 (2014). 24860161 10.1093/bioinformatics/btu354 PMC4155256

[b47] Ruzzene M., Di Maira G., Tosoni K. & Pinna L. A. Assessment of CK2 constitutive activity in cancer cells. Methods Enzymol 484, 495–514 (2010). 21036247 10.1016/B978-0-12-381298-8.00024-1

[b48] Bendall S. C. . Prevention of amino acid conversion in SILAC experiments with embryonic stem cells. Mol Cell Proteomics 7, 1587–1597 (2008). 18487603 10.1074/mcp.M800113-MCP200 PMC2556023

[b49] Arrigoni G. . Mass spectrometry analysis of a protein kinase CK2beta subunit interactome isolated from mouse brain by affinity chromatography. J Proteome Res 7, 990–1000 (2008). 18220339 10.1021/pr070500s

[b50] Nogales-Cadenas R. . GeneCodis: interpreting gene lists through enrichment analysis and integration of diverse biological information. Nucleic Acids Res 37, W317–322 (2009). 19465387 10.1093/nar/gkp416 PMC2703901

[b51] Szklarczyk D. . STRING v10: protein-protein interaction networks, integrated over the tree of life. Nucleic Acids Res 43, D447–452 (2015). 25352553 10.1093/nar/gku1003 PMC4383874

