# Supplementary material for: Generation and quantitative proteomics analysis of CK2α/α’(−/−) cells
Source: Sci Rep. 2017 Feb 17;7:42409. doi: 10.1038/srep42409 (PMC5314375; doi:10.1038/srep42409)
Supplement: Supplementary Figures and Table [file srep42409-s1.pdf]

SUPPLEMENTARY INFORMATION

Generation and quantitative proteomics analysis of CK2α/α’$^{(-/-)}$ cells

Christian Borgo[1, †], Cinzia Franchin[1,2, †], Stefano Scalco[2], Valentina Bosello-Travain[3], Arianna Donella-Deana[1], Giorgio Arrigoni[1,2,*], Mauro Salvi [1,*], Lorenzo A. Pinna [1,4,*]

[1]Department of Biomedical Sciences, University of Padova, Via U. Bassi 58/B, Padova, Italy

[2]Proteomics Center, University of Padova and Azienda Ospedaliera di Padova, via G. Orus 2/B, Padova, Italy

[3] Department of Molecular Medicine, University of Padova, Via Gabelli 63, Padova, Italy

[4]CNR Institute of Neurosciences, Via U. Bassi 58/B, Padova, Italy

*To whom correspondence should be addressed: Mauro Salvi, mauro.salvi@unipd.it, Giorgio Arrigoni, giorgio.arrigoni@unipd.it, Lorenzo A. Pinna, lorenzo.pinna@unipd.it

†These authors contributed equally to this work

CK2α/α’ KO clone A

A

Csnk2a1

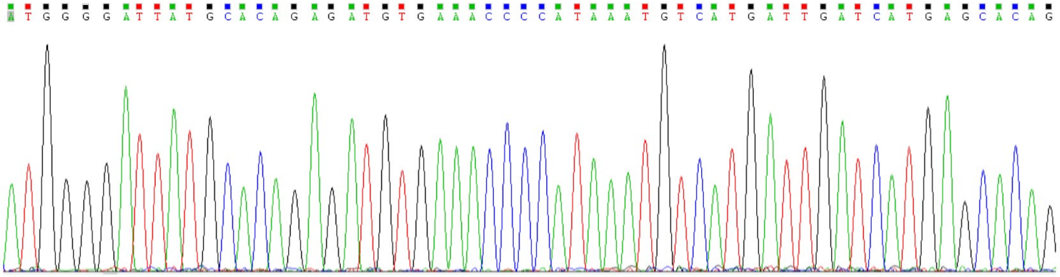

Cr Target site

```
wt_α    ATGGGGATTATGCACAGAGATGTGAAACCCCATAA-TGTCATGATTGATCATGAGCACAG
KOA_α   ATGGGGATTATGCACAGAGATGTGAAACCCCATAAATGTCATGATTGATCATGAGCACAG
        *********************************** ************************
```

B

Csnk2a2

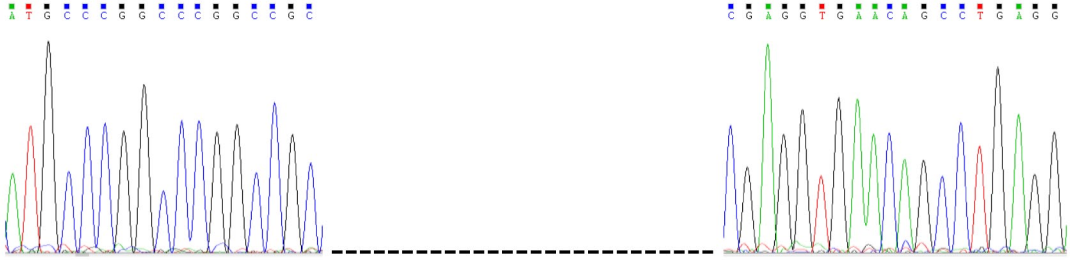

Cr Target site

```
WT_α′   ATGCCCGGCCCGGCCGCGGGCAGTCGGGCCCGGGTCTACGCCGAGGTGAACAGCCTGAGG
KOA_α′  ATGCCCGGCCCGGCCGC------------------------CGAGGTGAACAGCCTGAGG
        *****************                        *******************
```

Figure S1. Chromatogram from Sanger sequencing of purified PCR products amplified from genomic DNA spanning the crispr target sequence in *Csnk2a1* (CK2α) (A) and *Csnk2a2* (CK2α’) (B) genes in clone A. Mutation detected are observed to be the same on both alleles of each gene. The mutation consists of a 24 nucleotide deletion. Cr target site: crisper target site.

CK2α/α’ KO clone B

A

Csnk2a1

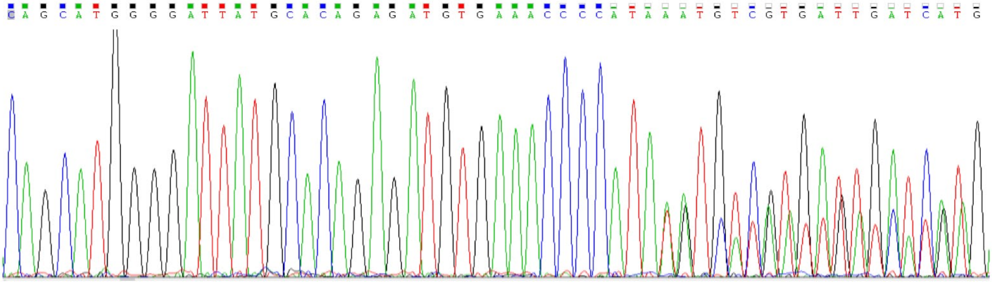

B

Csnk2a1 (allele 1)

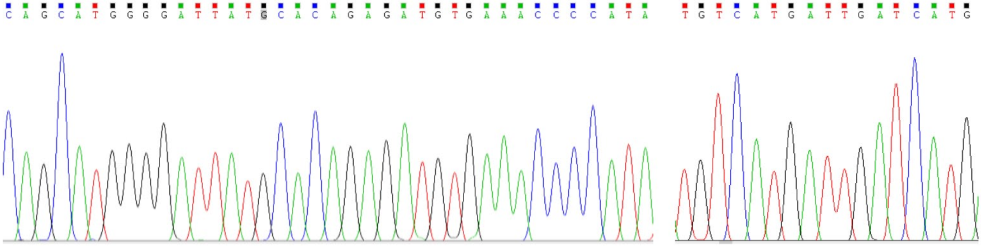

Cr Target site

```
wt_α    CAGCATGGGGATTATGCACAGAGATGTGAAACCCCATAATGTCATGATTGATCATG
KOB_α   CAGCATGGGGATTATGCACAGAGATGTGAAACCCCATA-TGTCATGATTGATCATG
        ************************************** *****************
```

Csnk2a1 (allele 2)

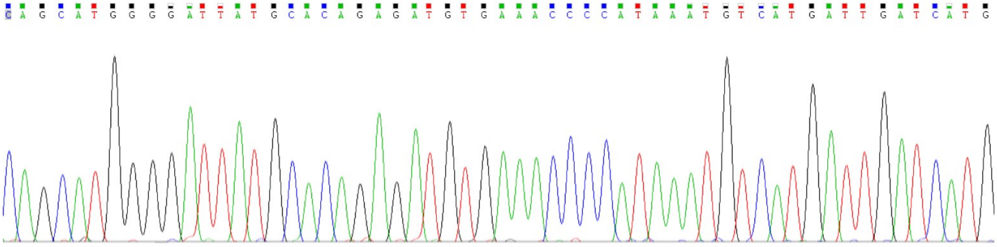

Cr Target site

```
wt_α    CAGCATGGGGATTATGCACAGAGATGTGAAACCCCATAA-TGTCATGATTGATCATG
KOB_α   CAGCATGGGGATTATGCACAGAGATGTGAAACCCCATAAATGTCATGATTGATCATG
        *************************************** *****************
```

CK2α/α’ KO clone B

C

Csnk2a2

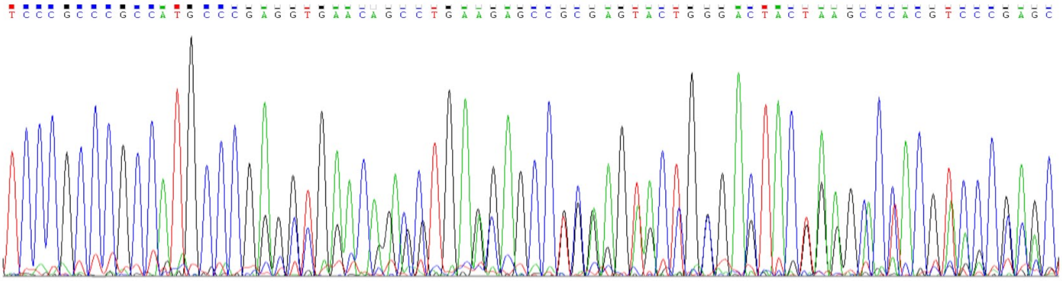

D

Csnk2a2 (allele 1)

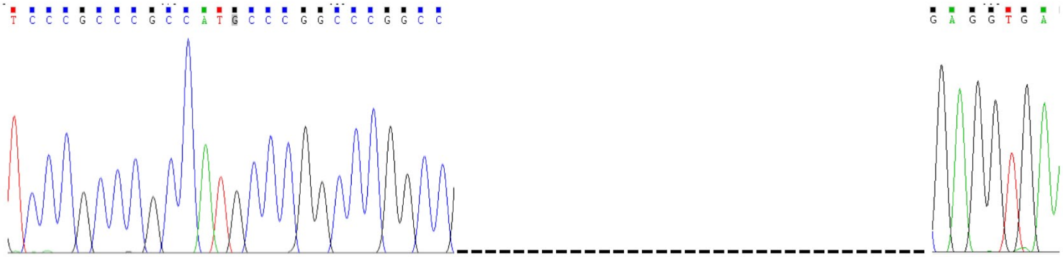

Cr Target site

```
wt_α′    TCCCGCCCGCCATGCCCGGCCCGGCCGCGGGCAGTCGGGCCCGGGTCTACGCCGAGGTGA
KOB_α′   TCCCGCCCGCCATGCCCGGCCCGGCC---------------------------GAGGTGA
         **************************                           *******
```

Csnk2a2 (allele 2)

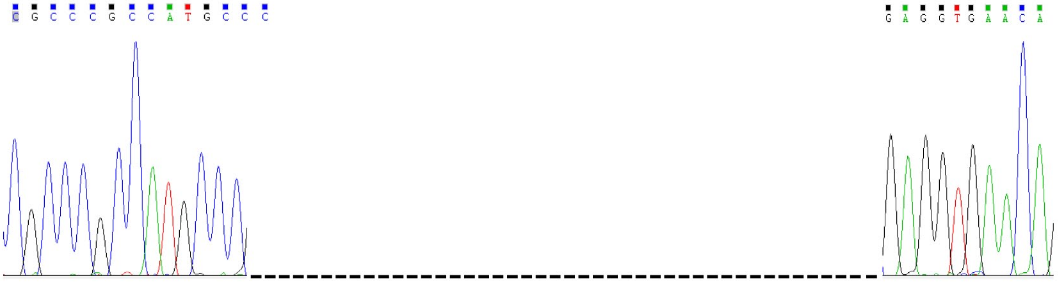

Cr Target site

```
wt_α′    CGCCCGCCATGCCCGGCCCGGCCGCGGGCAGTCGGGCCCGGGTCTACGCCGAGGTGAACA
KOB_α′   CGCCCGCCATGCCC------------------------------------GAGGTGAACA
         **************                                    **********
```

Figure S2. (A,C) Chromatogram from Sanger sequencing of purified PCR products amplified from genomic DNA spanning the crispr target sequence *Csnk2a1* (CK2α) (A) and *Csnk2a2* (CK2α’) (C) genes in clone B. (A,C) Mutation(s) are observed but allelic events are unclear. Cr target site: crisper target site. (B,D) Chromatograms representing the 2 different mutations identified from Sanger Sequencing after cloning PCR product into PGEMeasy vector, transforming into competent cells and selecting 8-10 single colonies from each clone. (B) For *Csnk2a1* allele 1 the mutation consist of a 1 nucleotide deletion and for allele 2 the confirmed mutation is 1 nucleotide insertion. (D) For *Csnk2a2* allele 1 the mutation consists of a 27 nucleotide deletion and for allele 2 the confirmed mutation is 36 nucleotide deletion.

CK2β KO

Csnk2b

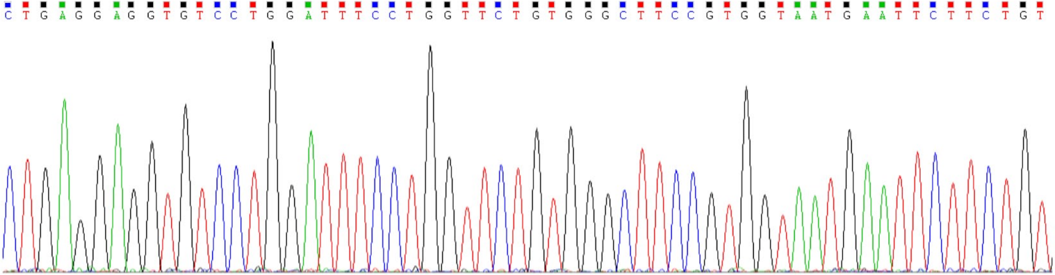

Cr Target site

```
wt_β    CTGAGGAGGTGTCCTGGATTTCCTGGTTCTGTGGCT-CCGTGGTAATGAATTCTTCTGT
KO_β    CTGAGGAGGTGTCCTGGATTTCCTGGTTCTGTGGCTTCCGTGGTAATGAATTCTTCTGT
        ************************************ **********************
```

Figure S3. Chromatogram from Sanger sequencing of purified PCR products amplified from genomic DNA spanning the crispr target sequence in *Csnk2b* (CK2β) gene. Mutation detected are observed to be the same on both alleles. Mutation consist of a 1 nucleotide insertion. Cr target site: crisper target site.

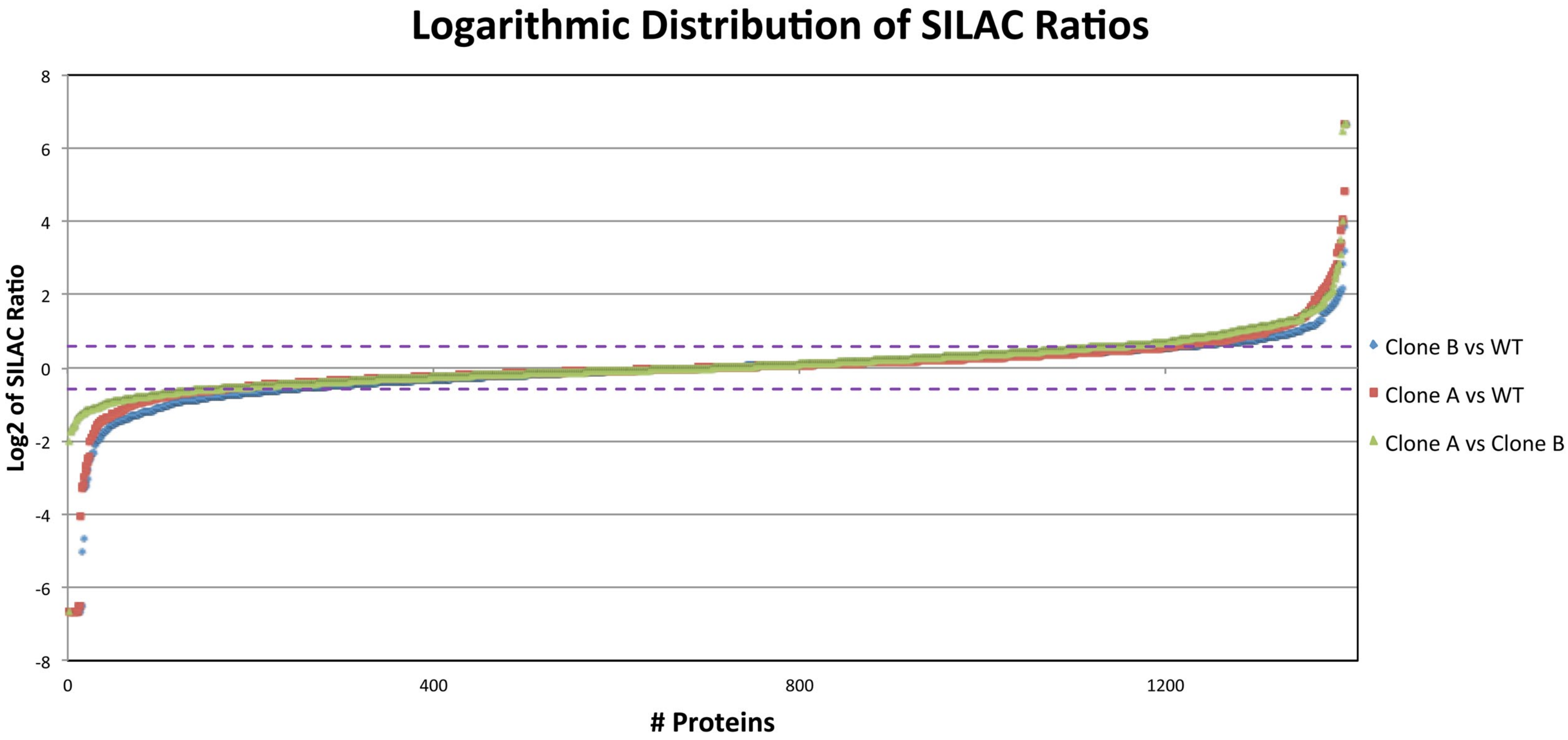

Figure S4. Logarithmic Distribution of SILAC Ratios.
The figure shows the logarithmic distribution of SILAC ratios for clone A vs WT (red curve), clone B vs WT (blue curve), and clone A vs clone B (green curve). Dotted lines indicate the value above which (0.58, corresponding to a Fold Change ≥ 1.5) or below which (-0,58, corresponding to a Fold Change ≤ -1.5) the proteins were considered as significantly altered.

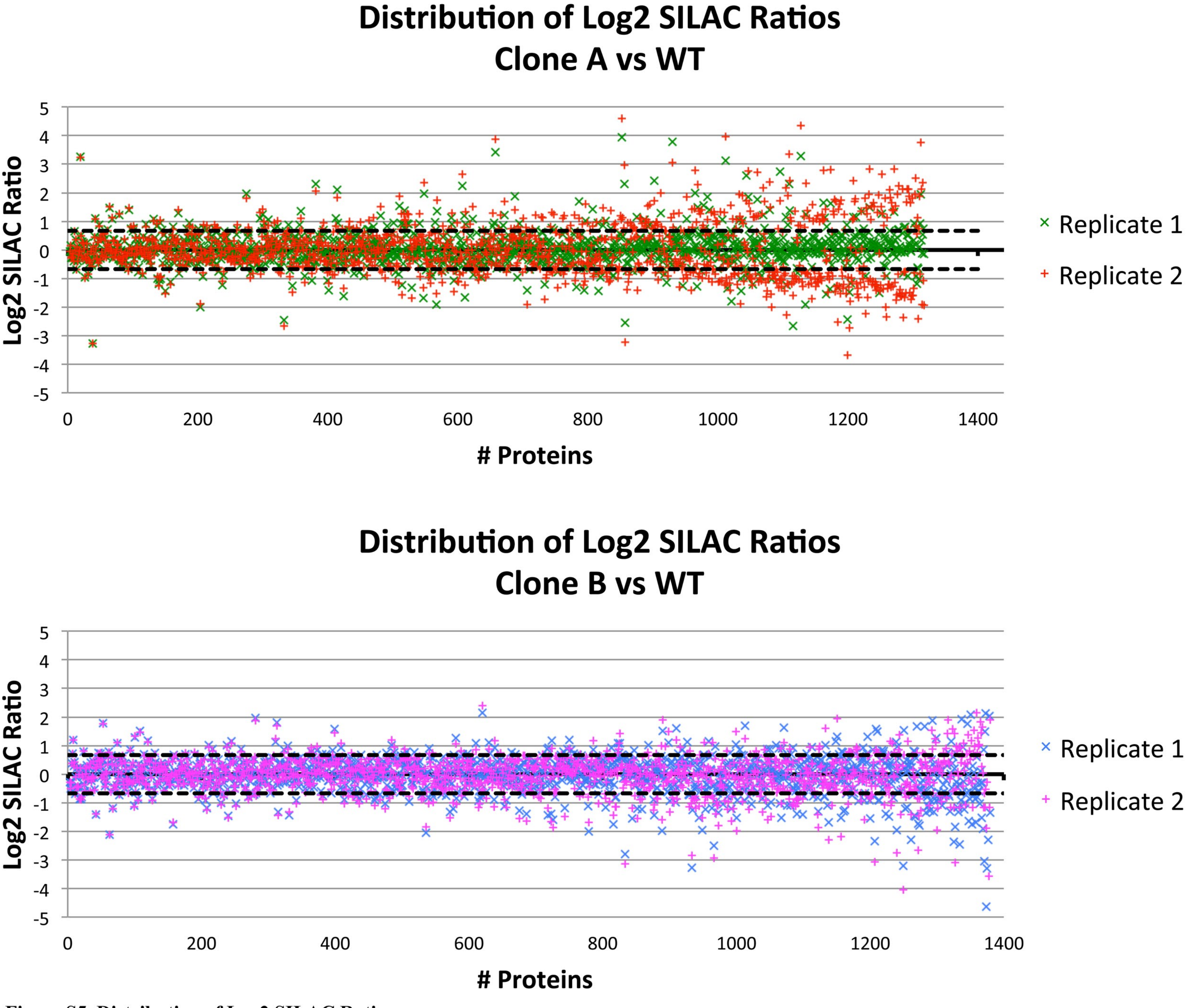

Figure S5. Distribution of Log2 SILAC Ratios.

The figure shows the distribution of SILAC ratios for clone A vs WT and for clone B vs WT, for both biological replicates. Dotted lines indicate the value above which (0.58, corresponding to a Fold Change ≥ 1.5) or below which (-0,58, corresponding to a Fold Change ≤ -1.5) the proteins were considered as significantly altered.

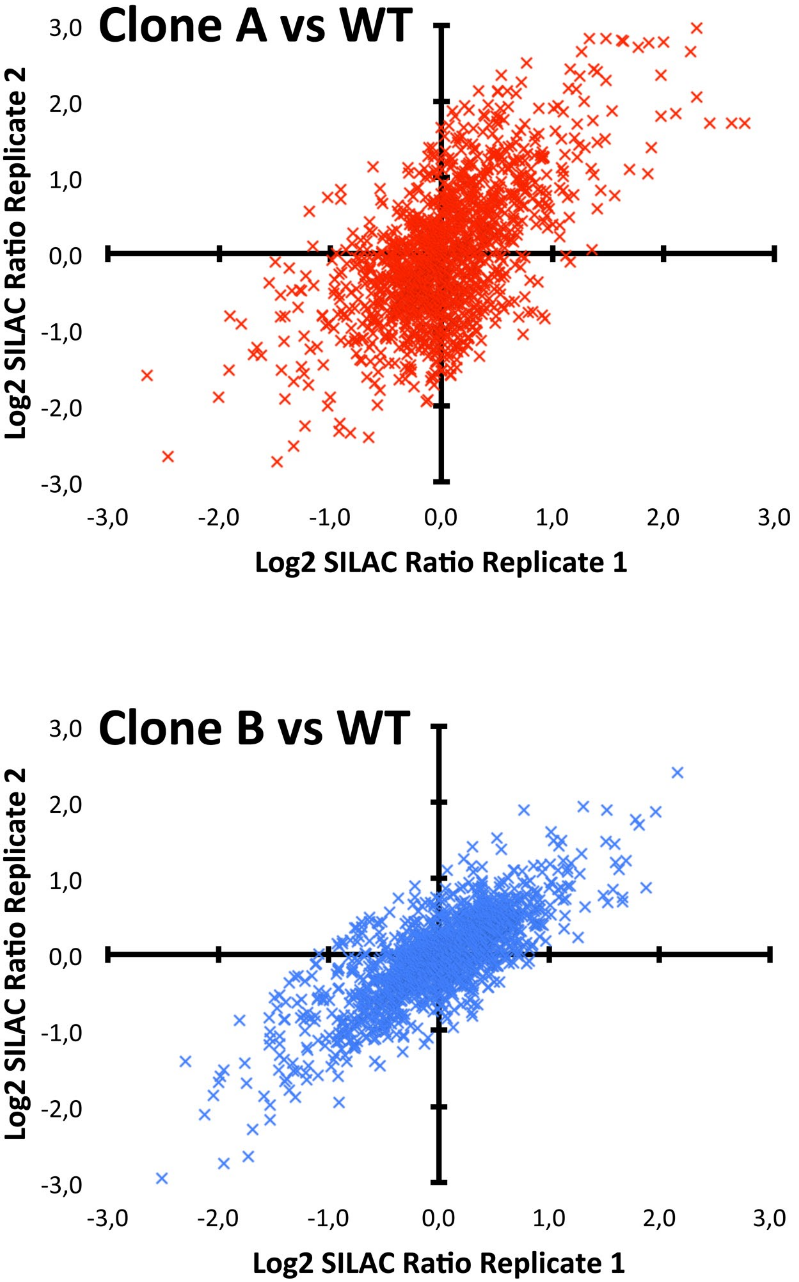

Figure S6. Reproducibility of quantification data across biological replicates. Panels show the reproducibility of SILAC ratios between the two biological replicates for clone A (upper) and B (lower).

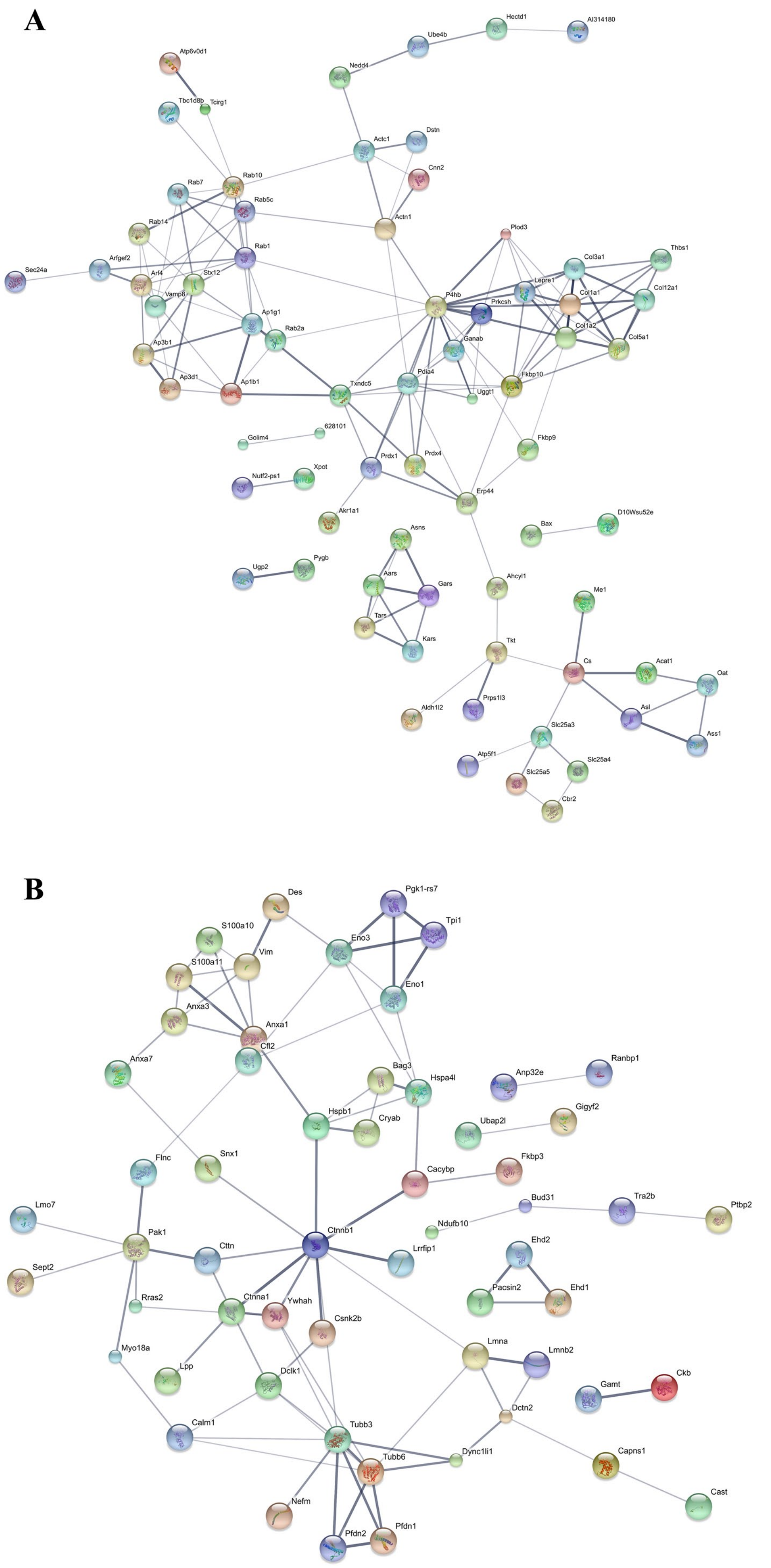

Figure S7. String Analysis

STRING analysis of proteins whose phosphorylation level is significantly affected (±50%) in CK2α/α'$^{(-/-)}$ clones. Disconnected nodes in the network have been hidden. Line thickness indicates the strength of data support.

SUPPLEMENTARY TABLE LEGENDS

Table S1. List of proteins altered by at least 50% in both CK2α/α'$^{(-/-)}$ clones.

The table lists all proteins that were found to be significantly altered (fold change ≥ 1.5 or ≤ -1.5 and p value ≤0.05) in both clones with respect to the WT and in both biological replicates. Uniprot accession code, protein description, average fold change and p values are reported.

Table S2. Ingenuity Canonical Pathways.

The table lists the Ingenuity Canonical Pathways (along with the –log(p value) and, if calculated, the associated z-score) that appear to be altered in cells lacking the catalytic subunits of CK2. In green are highlighted the most significant pathways (p value ≤ 0.01).

Table S3. Disease and Biofunctions.

The table lists diseases or functions annotation that are affected by the lack of CK2 activity. Categories, p value, z-score and predication of activation/deactivation state are also reported.
